# Precolonial Centralization, Koranic Education and School Participation in Nigeria

**DOI:** 10.12688/gatesopenres.16358.1

**Published:** 2025-08-27

**Authors:** Musiliu Adeolu Adewole

**Affiliations:** 1 Federal University of Technology Akure School of Management Technology, Akure, Ondo, 340001, Nigeria

**Keywords:** Precolonial Centralization, Koranic Education, School Participation, Religion

## Abstract

Several studies have documented the persistence of economic development outcomes across space and over long periods. Other studies have argued that the reversal of fortune has also occurred over time and space. Since different areas of current Nigeria were once under the rule of states with different degrees of political centralization and later investment in Koranic, this study seeks to explore whether areas or districts under a more centralized political system are more likely to participate in large-scale school expansion programs, such as the 1976 Universal Primary Education (UPE) and 1999 Universal Basic Education (UBE). To check for evidence of the reversal of fortune, we determine whether the degree of state centralization on school participation is more or less in areas that have large investments in Koranic education. OLS results show that while an index of state centralization has a positive and significant impact on enrolment in UPE and UBE programs, the effect is negative and statistically significant for those with heavy investment in Koranic education (measured by district fraction of 1914-46 cohorts with Koranic education). The results are robust to adding an extensive range of explanatory variables and specification tests. While the structure of the economy at the onset of Islamic activities in Nigeria may have made investment in Koranic education worthwhile, the contemporary world does not require Koranic education to make either regional or national advancement possible. Thus, there is a clear case of mismatch between the demands of modern economic life and the skills possessed by a large section. Thus, well-designed policies are required to address this mismatch and accelerate inclusive economic development.

## 1. Introduction

In recent years, the development economics and political economy literature has increasingly emphasized the role of historical institutions and cultural legacies in shaping contemporary development outcomes ( Acemoglu et al., 2001; Nunn, 2008; Michalopoulos & Papaioannou, 2013). One central theme in this literature is the persistence of precolonial state capacity—often proxied by political centralization, and its enduring influence on economic and social indicators such as infrastructure provision, human capital accumulation, and service delivery ( Gennaioli & Rainer, 2007; Besley & Persson, 2013; Lowes et al., 2017). At the same time, other studies highlight a “reversal of fortune”, wherein historically centralized or economically advanced regions today lag behind due to cultural, institutional, or colonial legacies that stifled adaptation to modern growth-enhancing institutions ( Acemoglu et al., 2002; Nunn & Wantchekon, 2011; Chaney, 2016).

This study contributes to this evolving discourse by investigating how the interaction between precolonial political centralization and investment in Islamic (Koranic) education has influenced modern school participation in Nigeria, particularly in the context of tuition-free, large-scale public education programs such as the 1976 Universal Primary Education (UPE) and the 1999 Universal Basic Education (UBE) programs. Despite the universal and free nature of these interventions, regional disparities in school participation persist—most notably, lower enrolment rates in Nigeria’s historically centralized northern regions, where Islamic institutions were dominant long before the colonial era.

Earlier works ( Archibong, 2019; Antoninis, 2014; Lincove, 2009) offer valuable insights into religious barriers to schooling and the limits of infrastructure-led interventions. However, they fall short of jointly exploring how precolonial political structures and religious educational investments interact to shape modern outcomes. This paper addresses that gap by examining whether historically centralized districts that also invested heavily in Koranic education exhibit different responses to public education programs compared to those without such overlapping legacies.

Our conceptual framework builds on the idea that historical institutions shape preferences and incentives ( North, 1990; Greif, 2006), and that religiously grounded educational systems, particularly Koranic schools, may foster cultural norms and social sanctions that discourage participation in Western-style formal education, even when it is free ( Fryer, 2007; Chaney, 2016). This framework is especially relevant in Nigeria, where colonial and post-colonial education policies have had uneven uptake due to varying degrees of alignment with local norms and religious worldviews ( Okoye & Pongou, 2024; Efobi, 2024).

To empirically test our hypotheses, I leverage district-level data on school enrolment from the 2006 Nigerian Census and historical measures of political centralization and Islamic education exposure drawn from the Murdock Ethnographic Atlas and archival records. Our econometric approach includes interaction terms between state centralization and Koranic education, and extensive controls for geography, demography, colonial missionary activity, natural resources, and conflict exposure. I also perform multiple robustness checks, including IV regressions using colonial restrictions on missionary activity as an instrument for koranic education intensity.

Our results reveal that while precolonial centralization alone is positively associated with school enrolment, this effect is significantly attenuated or reversed in areas with high historical investment in Koranic education. This pattern holds across both UPE and UBE cohorts and is robust to alternative specifications and controls. These findings suggest a nuanced mechanism where historical political complexity can either promote or hinder development, depending on its interaction with cultural-religious legacies. In so doing, the study contributes to the broader literature on persistent and path-dependent development outcomes, and the role of cultural resistance in shaping human capital trajectories in Sub-Saharan Africa.

The remainder of the paper is structured as follows: Section 2 provides a historical overview of Islamic and Koranic education in Nigeria. Section 3 describes the data sources and key variables. Section 4 outlines the empirical strategy. Section 5 presents the main results and robustness checks. Section 6 concludes with implications for policy and further research.

## 2. History of Islamic and Koranic Education in Nigeria

### 2.1 Islam and Islamic Education in Nigeria ^1^

Islam first reached the Savannah region of western Africa around the 8th century A. D, some 1000 years before Christian missionaries first set their foot on the shores of Nigeria. The spread of Islam in this region has brought about extensive commercial relationships with North Africa ( Fafunwa, 1991). With the established religious and trade relationship between the savannah region of West Africa and North Africa, new cultural experiences, which included the development of new intellectual and literacy skills that later made the region the hub of intellectual activities for many centuries, began to emerge.

Through the efforts of Islamic cleric and scholar Hamed Muhammed Mani, Islam was introduced to the ruler of Kanem Bornu between 1085 and 1097 ( Fafunwa, 1991). From Kanem-Bornu, traders and scholars took Islam to Hausa Land in the early 14th century during the reign of Ali Yaji. From 1452 to 1463, books on Islamic theology and jurisprudence were introduced to Hausaland by Fulani scholars ( Fafunwa, 1991). During the 1463-99 reign of Muhammed Rumfa in Kano, Islam became firmly established, and many Islamic learning centers were established. Islamic scholars from Timbuktu also taught and preached Islam in Kano. Islam later spread to Katsina in the 15th century, with Katsina receiving pilgrims from Mecca and Timbuktu scholars, with latter groups from Timbuktu often visiting books on Islamic divinity and etymology.

From Timbuktu, Islam was introduced to Yorubaland towards the close to the 19th century ( Johnson, 1966), although before 1627, this area was already known to Muslims, when Ahmed Baba identified Yorubaland as a location where paganism was prevalent and Islam had no root ( Fafunwa, 1991). Learned Islamic scholars formally brought Islam to Yorubaland through lorin in 1830. Some of these scholars made contact with Ibadan, about the time the town was established, and later with Ijebu-Ode and Abeokuta. Ilorin became a center of Islamic learning, with many basic and advanced schools established to teach Islamic religion. Ilorin often received famous Islamic scholars from Katsina, Kano, and the south-west. With the passage of time, Ibadan became a renowned center of excellence in Islamic education, with the construction of many mosques and Islamic schools to encourage the spread of Islam.

## 3. Data Sources and Description

### 3.1 School Enrolment Data

Our dependent variables, the fraction of UPE and UBE cohorts reported to have enrolled in schools, were taken from the district level summary of Nigeria’s 2006 census, which contains relevant information. We restricted ourselves to cohorts who, by virtue of age, should have participated in the universal primary or basic education program. For the UPE cohorts, this included those reported to be in the 35-39 and 40-44 age brackets. Similarly, those in the 6-9 and 10-14 age bracket for the UBE cohorts were included in this study. The choice of these cohorts is primarily due to the fact that post-1970, the number of schools more than doubled ( Archibong, 2019). The number of schools remained unchanged at about 35,000 until 1989, and again increased to about 69,979 ( Archibong, 2019) by 2010, a period of 11 years after the re-introduction of UPE as UBE, with basic education becoming free and compulsory for the first time. The slash in the cost of education, coming from the direct reduction in tuition fees and access to schooling facilities, was required to increase educational attainment.

### 3.2 Explanatory Variables

Murdock’s (1967) Ethnographic Atlas is the source of state centralization and other relevant historical data. We matched Murdock’s ethnographic data using the information available in Archibong (2019), who identified which of the historical variables could be matched to the 774 districts or LGAs. The Atlas provides descriptions of the spatial distribution of ethnic groups across Africa, and for much of Africa, the data capture the pre-colonial attributes of these ethnicities, although they were sampled in the 20th century ( Murdock, 1967; Michalopoulos, 2012; Obikili, 2016). Murdock constructed historical variables and the political complexity of each ethnic group using both primary and secondary sources, including other 59 historical variables besides the political complexity of state centralization. Murdock’s 243 ethnic locations out of 1265 fall within the African continent and 117 are currently within Nigeria. At the most, over three ethnic groups would overlap a given district today; they tend to share the same level of state centralization or political complexity value.

Following Gennaioli and Rainer (2007), Michalopoulos (2012), Alesina et al. (2013), and Archibong (2019), we adopted Murdock’s (1967) information on “Jurisdictional Hierarchy beyond the Local Community Level”. This measure ranges from zero to four, starting from the absence of hierarchical authority to four levels or layers of authority. This measure describes the complexity of political systems that allow pre-colonial states to deliver on the provision of social services and collect taxes.

For our benchmark model, we choose additional historical ***variables*** depicting various degrees of political and economic development. First, the Ethnographic Atlas describes the complexity of human settlement, namely nomadic or fully migratory, semi-nomadic, semi-sedentary, compact but impermanent settlement, neighborhood of dispersed family homesteads, separated hamlets forming a single community, compact and relatively permanent settlements, and complex settlements. The values range from 1 to 8 in order of complexity. Second, ethnographic variable 40 depicts the use of large animals by ethnic groups inhabiting the 774 districts in Nigeria. The ***seven categories are*** the absence or near absence of large domestic animals (1); pigs are the only large domestic animals (2); sheep and goats without large domestic animals (3); equine animals such as horses and donkeys (4); deer (5); camels, alpacas, or llamas (6); and bovine animals and cattle, Mithun, water buffalo, and yaks (7). The variable for the use of large animals was constructed by assigning 1 to locations with ethnic groups that fell within categories 2 to 7 and 0 otherwise.

Third, we used an indicator variable constructed from the Murdock Ethnographic Atlas v39, indicating whether groups inhabiting any district in our sample were using animal plow at the time of observation or whether plow existed before contact. Fourth, the historical variable indicates the intensity of the agricultural practices. This was obtained from v28 of the Murdock Ethnographic Atlas, with the following categories of intensity: no agriculture, casual agriculture, extensive or shifting agriculture, horticulture, intensive agriculture, and intensive irrigated agriculture. It ranges from 1 to 6, with higher values indicating a greater intensity.

The final historical variables in our baseline specification were the extent to which districts had groups that mainly hunt and herd large animals as a form of subsistence. The hunting variable taken from v2 and the herding variable are from v4 of the Murdock Ethnographic Atlas. The level of dependence for hunting and animal husbandry is graded as 0-5 %, 6-15%, 16-25%, 26-35%, 36-45%, 46-55%, 56-65%, 66-75%, 76-85% and 86-100%.

**More Historical Variables**

Because the present-day level of school enrolment could have been influenced by contact (or lack thereof ) with Europeans, we introduced several historical variables that reflect this experience and could serve as confounding factors if not accounted for.

We included a variable measuring the number of Christian mission stations (Protestant and Catholic) per square kilometer as of 1924 for each of the 774 districts. Alternatively, we introduced a variable measuring the number of mission schools in 1925. Data originally provided by Century Company (1911) and used by Nunn and Wantchekon (2011) ^2^ *to construct a dummy variable assigning 1 to every district which had a European explorer passing through it, otherwise zero.* Using the century map again, we constructed an indicator variable for every district through which the colonial rail passed.

**District Geographic Controls**

In addition to the variables listed in our benchmark specifications, we introduce several geographic controls. First, we used the district terrain ruggedness index obtained by Nunn and Puga (2012). This geographic variable measures the extent to which land can be worked upon, or the extent to which it facilitates or impedes access to certain localities, including schools and other public facilities.

The second was the extent of temperature experienced in a district. It was constructed by including temperatures at the time of birth for the UPE and UBE cohorts in our specification. The data were obtained from GeoAid ( Goodman et al., 2019). This is because drought, prompted by high temperatures and water shortages, can impede several activities ( Maccini and Yang, 2009), including participation in formal education. Third, we constructed district rainfall variables for UPE and UBE cohorts in the same way as we did for the temperature variable. Fourth, we included the presence or absence of gemstones (21 types) in each of the 774 districts. The presence of gold and crude oil was also included in the extended econometric specifications. Fifth, various dimensions of conflict were added to our specification because conflict can affect enrolment (for UBE because of the data used).

Sixth, the distance variables that can affect school enrolment are included in our augmented specifications. This includes the mean distance to the coast, water bodies, roads, country borders, major cities, lootable gold locations, gemstone deposit areas, drug cultivation, and onshore crude oil exploration sites ^3^. The summary statistics for the dependent variables and the selected baseline variables are presented in Table 1.

**Table 1.** Descriptive statistics: Dependent variables and baseline variables.

Variable description	OBS.	Mean	Std. Deviation	Minimum	Maximum
Enrolment Rates for UBE Cohorts Aged Between 6-9 Years in 2006	774	46.7405	22.56239	0.8398606	95.2381
Enrolment Rates for UPE Cohorts Aged Between 35-39 Years in 2006	774	61.69573	23.27299	6.899872	97.14136
Jurisdictional hierarchy beyond local community as measure of state centralization	774	2.219638	1.245256	1	4
District complexity of settlement pattern	774	5.731266	2.364601	0	8
District ever used plough, before and after contact with Europeans	774	0.9069767	0.2906528	0	1
Degree of dependence on hunting	774	0.4082687	0.51496	0	2
Intensity of Agriculture	774	3.255814	1.383393	0	6
District population per square km in 1975	774	428.3484	1328.088	0	14209.84

This table showed the summary statistics at the Local Government Area (LGA).

## 4. Empirical Model

To determine the separate or interactive effects of state centralization and Koranic education on school enrolment of the UPE and UBE cohorts, we adopted the Archibong (2019) model to determine the relationship between each of these important explanatory variables and observed school outcomes. Model 1 captured the essence of the proposed approach.

$$S_{\mathrm{ds}}=\beta_{0}+\lambda_{1}S{\text{Centra}}_{d}+\lambda_{2}{\mathrm{Kor}}_{d}+\lambda_{3}\left(S{\text{Centra}}_{d}\right)\left({\mathrm{Kor}}_{d}\right)+X_{d}^{h}\alpha +S_{\mathrm{fe}}+\varepsilon_{\mathrm{ds}}$$

The dependent variable ( $S_{\mathrm{ds}}$ ) is the school enrolment rate for UPE cohorts (born after 1969) and UBE cohorts (born after 1992). ${\mathrm{Centra}}_{d}$ is our measure of district historical state centralization index. ${\mathrm{Kor}}_{d}$ represents the district fraction of 1916-46 cohorts having Koranic education. The key variable of interest is the interaction variable [ $\left(S{\mathrm{Centra}}_{d}\right)\left({\mathrm{Kor}}_{d}\right)$ ], which allows us to determine the impact of state centralization on school enrolment when a district has two standard deviations higher investments than other districts. $X_{d}^{h}$ is a vector of baseline historical variables reflecting the district complexity of human settlements, use of large domestic animals, use of animal-drawn plows, and the degree of dependence on hunting and herding. Other historical variables were added to check the robustness of the estimates of our key explanatory variable of interest $\left(S{\mathrm{Centra}}_{d}\;X\;{\mathrm{Kor}}_{d}\right)$ and are included alongside other district-level geographic variables that will be added to the Model 1 specification later. The $X_{d}^{g}$ vector of district geographic variables was also added as a robustness check to our main results. $\;S_{\mathrm{fe}}$ is state fixed effects and should capture time-invariant factors operating at the state level that affect enrolment. We addressed spatial autocorrelation issues by clustering at the state level. Ordinary Least Squares (OLS) is used throughout, and because of endogeneity issues, we do not claim that our regression results represent a causal relationship between each of the key explanatory variables and school enrolment rates.

## 5. Discussion of Results

In starting our regression analysis based on Model 1, we separately include the three key variables of interest: pre-colonial centralization, the Koranic education dummy, and the interaction of pre-colonial centralization and Koranic education variables. Table 2 (column, Panels A and B) includes only centralization for the UBE and UPE cohorts, respectively. The estimate shows a strong and statistically significant relationship between pre-colonial centralization capacity and school enrolment for UBE cohorts (Panel A). That is, the 1976 tuition-free primary education program, which started with a substantial reduction in access costs due to large-scale school construction, had greater effects in districts with historical exposure to state centralization. That is, residents in districts that were once in highly centralized states are more likely to enrol those in districts with less centralized status.

**Table 2.** OLS regression: Dependent variable: Enrolment rate.

Panel A 6-9 Cohorts	All	All	All	All	All	All	All
State Centralization Beta Coefficient	4.7211 ^a^ (0.5610) 0.2606 ^a^			5.6998 ^a^ (0.5908) 0.3156 ^a^	5.8077 ^a^ (0.7344) 0.3216 ^a^	5.0429 ^a^ (0.7170) 0.2792 ^a^	5.4496 ^a^ (0.7233) 0.3017 ^a^
Koranic Education Beta Coefficient		-41.8017 ^a^ (1.7456) -0.6355 ^a^		-28.2092 ^a^ (3.0730) -0.4289 ^a^	-23.5352 ^a^ (3.6201) -0.3578 ^a^	-24.366 ^a^ (3.4914) -0.3705 ^a^	-21.9052 ^a^ (3.5734) -0.3330 ^a^
(State Centralization) (Koranic Education) Beta Coefficient			-0.1004 ^a^ (0.0072) -0.4153 ^a^	-0.0575 ^a^ (0.0119) -0.2379 ^a^	-0.0633 ^a^ (0.0142) -0.2619 ^a^	-0.0626 ^a^ (0.0136) -0.2587 ^a^	-0.0674 ^a^ (0.01374) -0.2786 ^a^
Historical Variables					Yes	Yes	Yes
1975 LGA Population Per Square Km						Yes	Yes
State Fixed Effects							Yes
F-Statistics	70.83	573.46	192.69	255.36	120.80	121.47	111.97
Observations	774	771	771	771	771	771	771
R-Squared	0.0679	0.4039	0.1725	0.4548	0.4796	0.5269	0.5346
Panel B 35-39 Cohorts							
State Centralization Beta Coefficient	4.3496 ^a^ (0.5932) 0.2327 ^a^			4.5277 ^a^ (0.6537) 0.2433 ^a^	5.1150 ^a^ (08111) 0.2749 ^a^	4.2618 ^a^ (07913) 0.2290 ^a^	4.7931 ^a^ (0.7946) 0.2576 ^a^
Koranic Education Beta Coefficient		-41.0934 ^a^ (1.8787) -0.6064 ^a^		-32.2812 ^a^ (3.3923) -0.4764 ^a^	-25.8258 ^a^ (3.9057) -0.3811 ^a^	-26.7525 ^a^ (3.7584) -0.3948 ^a^	-23.5386 ^a^ (3.8075) -0.3474 ^a^
(State Centralization) (Koranic Education) Beta Coefficient			-0.0958 ^a^ (0.0082) -0.3845 ^a^	-0.0356 ^a^ (0.0139) -0.1428 ^a^	-0.0472 ^a^ (0.0158) -0.1896 ^a^	-0.0464 ^a^ (0.0151) -0.1862 ^a^	-0.0527 ^a^ (0.0150) -0.2113 ^a^
Historical Variables					Yes	Yes	Yes
1975 LGA Population Per Square Km						Yes	Yes
State Fixed Effects							Yes
District Fixed Effects							
F-Statistics	53.76	478.44	135.43	196.45	98.06	99.26	93.55
Observations	774	771	771	771	771	771	771
R-Squared	0.0542	0.3677	0.1478	0.4014	0.4323	0.4879	0.5002

OLS method is used in the regressions reported here. Standard errors indicated in parenthesis are clustered at the district level. District (or LGA) is the unit of observation. CS is the enrolment rate for children who in 2006 were in the 6-9 age category. Historical variables include complexity settlement patterns, use of animal plough, intensity of agriculture and degree of dependence on hunting.

^a^ Significant at 1% level.

^b^ Significant at 1% level.

^c^ Significant at 1% level.

The regression estimates showed that a unit increase in centralization increases UBE and UPE school enrolment by 17.31 and 16.18 percent respectively, and the estimates are statistically significant at the 1% level. Alternatively, one standard deviation increase in centralization leads to 0.3438 increases in UBE cohorts’ school enrolment and 0.31 for UPE-cohort school enrolment measure. In Column 2, the Koranic education variable was added to the model. The effect of 1916-46 cohorts’ Koranic education was negative and statistically significant, reducing school enrolment by approximately 29.6 and 29.7 percent for the UBE and UPE cohorts, respectively. Better put, a standard deviation increase in Koranic education leads to a reduction in school enrolment by 0.64 and 0.6141 for UBE and UPE, respectively. In column 2, the coefficient of determination is higher than that shown in the Column 1 regression. This implies that Koranic education has greater explanatory power than the centralization variable in explaining the variation in the school environment. The more important interaction variable included in Column 3 is also strong and negative, indicating that residents in highly centralized districts with heavy investment in Koranic education are less likely to be enrolled in school, which is true for both the UBE and UPE cohorts. The estimate is 22 % lower for school enrolment rates for both UBE and UPE cohorts relative to districts with less centralized state structure and less Koranic education. The estimate was statistically significant at 1%.

In Column 4, we added three 3 variables to the specification simultaneously. As shown in Panels A and B of Table 1, all pre-colonial centralization, Koranic education, and the interaction variable are still statistically significant at 1% with only the interaction variable for UPE cohorts being significant at 10% (Panel B Table 2). With the three variables added simultaneously, the explanatory power of our model increases, with Koranic education and the interaction variable giving it greater explanatory power.

The strong negative relationship between centralization and the Koranic education variable and school environment indicates that the reversal of economic fortune can occur when critical intervention of great importance takes place. This is in line with the reversal of fortune documented in Acemoglu, Johnson and Robinson (2002), although contested by Putterman and Weil (2010). The results presented here are in sharp contrast to the findings in the literature on the relationship between state centralization and development outcomes ( Gennaioli and Rainer, 2007). Negative reversal of fortune has also been associated with the African slave trade ( Nunn, 2008; Nunn and Wantchenkon, 2011) and Tsetse prevalence and the rapid growth in Islamic literature relative to scientific works ( Chaney, 2013, 2016). There has also been a positive reversal of fortune from well-implemented health and education programs that has led to significant improvements in socioeconomic status ( Bleakley, 2007, 2010; Lucas, 2010; Cutler, Fung, Kremer, Singhal and Vogl, 2010; Feyrer, Politi and Weil, 2017; Ager, Hansen and Jensen, 2018; Duflo, 2001). Altogether, these three variables can explain the large change in school enrolment for both the UPE and UBE cohorts.

Because districts governed under highly centralized systems might also be more developed along other dimensions, we introduce Murdock’s historical variables in Column 5, depicting the complexity of its settlement system (use of animal plow, herding, hunting intensity, and intensity in agricultural practice). The relevant estimate was not dramatically changed by the inclusion of these variables, with the interaction increasing slightly. The population density variable, 1975 district population for the UPE cohorts, and 1990 district population density for the UBE cohorts are included in the Column 6 regression; however, neither the Koranic education nor the centralization-Koranic education variable change in terms of statistical and economic significance, although the estimate dropped slightly. Introducing state fixed effects did not significantly change the estimates (Column 7).

Overall, district pre-colonial centralization increases school enrolment significantly in specifications that include all over baseline variables, both for the UPE and UBE cohorts. Similarly, a higher level of investment in the Koranic education report reduced school enrolment for the UPE and UBE cohorts. If we consider the mean values of the dependent variables relative to the regression estimates of pre-colonial state centralization, Koranic education, and interaction variables, they are quite considerable when considered separately or in combination.

### 5.1 Robustness Tests: Sample Selection and Influential Observations

Although the estimates of the three key explanatory variables are statistically significant at 1 % with baseline control variables, there are concerns that specification issues due to influential observations from these explanatory variables might be at play. Therefore, we ran regressions that accounted for the presence of extreme values. Table 3 presents the results. First, we eliminate districts with a centralization index greater than three (four are at the highest level). Second, we removed districts with a centralization index of less than one (zero is the lowest) and those with a centralization index greater than three. Third, we exclude districts with no investment in Koranic education. Fourth, we also removed districts with more than two-thirds of the 1914-1946 district cohorts with Koranic education. The results for both the UPE and UBE cohorts are reported in Table 3. Columns 1-8 show that most estimates are economically and statistically significant for the three key variables.

**Table 3.** Robustness checks: Sample selection & influential observations.

Dependent variable: Enrolment rate								
Panel A Cohorts	6-9	35-39	6-9	35-39	6-9	35-39	6-9	35-39
Exclude Sample	Centralization>3	Centralization>3	v33>0 & v33<4	v33>0 & v33<4	LGA_KOR46<0	LGA_KOR46<0	LGA_KOR46>0.627	LGA_KOR46>0.627
State Centralization Beta Coefficient	4.0910 ^a^ (1.1673)	3.2296 ^b^ (1.28811)	3.3575 ^b^ (1.3921)	2.014457 (1.5098)	7.2322 ^a^ (1.0922)	7.1664 ^a^ (1.2785)	5.4325 ^a^ (0.7643)	4.9063 ^a^ (0.8229)
Koranic Education Beta Coefficient	-14.962 ^a^ (3.6470)	-15.2723 ^a^ (3.9017)	-13.924 ^b^ (6.9696)	-19.7625 ^a^ (6.7736)	-10.102b (4.3857)	-8.4539c (4.9438)	-43.5744a 7.735804	-52.0093a (8.3942)
(State Centralization) (Koranic Education) Beta Coefficient	-0.1060 ^a^ (0.0164)	-0.0968 ^a^ (0.0175)	-0.1095 ^a^ (0.0280)	-0.0783 ^a^ (0.0274)	-0.0776 ^a^ (0.0165)	-0.0683 ^a^ (0.0186)	-0.0838 ^a^ (0.0310)	-0.0729 ^b^ (0.0346)
Historical Variables	Yes	Yes	Yes	Yes	Yes	Yes	Yes	Yes
1975 LGA Population Per Square Km	Yes	Yes	Yes	Yes	Yes	Yes	Yes	Yes
District Fixed Effects	Yes	Yes	Yes	Yes	Yes	Yes	Yes	Yes
F-Statistics	91.10	91.70	77.53	78.27	68.06	51.30	68.36	58.47
Observations	621	621	537	537	480	480	599	599
R-Squared	0.5562	0.5533	0.5504	0.5569	0.5079	0.4355	0.4985	0.5013

Notes: Same as Table 2.

We used more standard methods to account for the influential observations. First, we used Student-T residuals that eliminated observations with plus or minus 2.0, 2.5, and 3.0. Second, we used Cook-Distance (based on d=4/771) to remove influential observations. Appendix Table 1 reports the results of the estimates. All estimates are economically significant and are statistically significant at *1%.* Furthermore, we used the DFIT and DFBETA ^4^ methods to check for outlying observations. Appendix Table 2 (columns 1-4) indicate that the presence of influential observations has no perceptible effects on our dependent variables. Based on RREG and QREG econometric methods ^5^, We ran regressions that corrected for influential observations based on the RREG and QREG econometric methods. Again, Appendix Table 2 (columns 5-8) show that estimates for the three variables barely changed, remaining economically and statistically significant.

Finally, we used dummy variables for the three core variables. The Koranic education dummy was constructed by assigning 1 to districts with equal or higher mean values (lga_kor46 ≥ 0.3), otherwise zero. For the state centralization index dummy, we assigned 1 to a district higher than one level of political authority (v33 ≥ 2). The interaction variable was constructed using the product of the Koranic and State Centralization dummies. The results were all significant (results not shown, but available).

### 5.2 Additional Explanatory Variables ^6^

Several covariates were included in our econometric specification to mitigate omitted variable bias. The included variables accounted for European influence in Appendix Table 3 (columns 1-4), conflict in Panels A and B columns 5-9 Appendix Table 3, rainfall and temperature (Appendix Table 4, Panels A and B), natural resources ***(*** Appendix Table 4, Panels A and B column 3), ***Chinese foreign aid*** (Appendix Table 4.0, Panels A & B Column 4), distance to centers of economic activity (Appendix Table 4 Panels A and B column 5), d ***iseases (*** (Appendix Table 4, Panels A and B column 6), with these additional explanatory variables, running regressions separately for male and female folks (Appendix Table 4, Panels A and B columns 7-8) and UBE cohorts in the 10-14 (Column 9) age category did not change results in any materially significant way.

### 5.3 Additional Robustness Checks

So far, the regression results from our baseline model and robustness checks have shown that state centralization has negative effects on school enrolment in districts where Koranic educational investment has been well above the average. The results still hold when many explanatory variables that could impact school enrolment are included in our econometric model. As we are not sure that our model is correctly specified, it is possible that one or two explanatory variables omitted from the econometric specification are strong enough to undermine the observed empirical relationships. In other words, our reported regression estimates may be biased by unobservables that are strongly correlated with our key explanatory variables, as well as school enrolment. In this concluding section (reported in Table 4), we attempt to perform a few more tests to mitigate the potential effects of unobservables on the observed results.

**Table 4.** Dependent variable: School enrolment OLS regression: More robustness checks.

Panel A 6-9 Cohorts	6-9 Cohorts	34-39 Cohorts	6-9 Cohorts	34-39 Cohorts	6-9 Cohorts	34-39 Cohorts	6-9 Cohorts	34-39 Cohorts	6-9 Cohorts	34-39 Cohorts
State Centralization Beta coefficient	1.9410 ^a^ (0.0224) {0.1056}	1.9443 ^a^ (0.0242) {0.1032}	4.3250 ^a^ (0.0417) {0.3374}	3.4895 ^a^ (0.0469) {0.2427}	2.4566 ^a^ (0.0546) {0.1402}	2.6128 ^a^ (0.0555) {0.1496}	-------	-------	--------	--------
(State-Centralization) (Koranic Education) Beta coefficient	-0.0287 ^a^ (0.0004) {-0.1171}	-0.0186 ^a^ (0.0004) {-0.0740}	-0.0567 ^a^ (0.0006) {-0.3665}	-0.0354 ^a^ (0.0007) {-0.2042}	-0.0398 ^a^ (0.0012) {-0.1683}	-0.0283 ^a^ (0.0012) {-0.1200}	-------	-------	--------	--------
Koranic Education Beta coefficient	-10.9871 ^a^ (0.1095) {-0.1626}	-10.9243 ^a^ (0.1179) {-0.1577}	-3.4421 ^a^ (0.1410) {-0.0688}	-5.6163 ^a^ (0.1569) {-0.1001}	-27.2257 ^a^ (0.3161) {-0.4032}	-24.1253 ^a^ (0.3227) {-0.3585}	-37.570 ^a^ (0.0894) {-0.5293}	-10.462 ^a^ (0.1193) {-0.1944}	-31.290 ^a^ (0.0945) {-0.4308}	-8.958 ^a^ (0.1217) {-0.1540}
F-Statistics	32152.44	39082.08	5723.06	7770.20	3685.88	4881.62	18880.28	7726.73	24346.34	11333.67
Observations	527,094	527,094	268,578	268,578	84,990	84,990	388,548	138,546	388,548	138,546
Adj. R-Squared	0.7454	0.7099	0.5003	0.4807	0.6708	0.6476	0.6763	0.7150	0.6369	0.7105

OLS method is used in the regressions reported here. Standard errors indicated in parenthesis are clustered at the state level. District (or LGA) is the unit of observation. CS is the enrolment rate for children who in 2006 were in the 6-9 age category. Columns 1 and 2 include control for northern Nigeria dummy. All variables added to Appendix Table 4 regressions are included here. Columns 3 and 4 is exclusively for northern Nigeria sample only. Regressions in columns 5 and 6 are restricted to pairs of LGAs within 200km of each other. In columns 7-10, we ran for high (columns 7 and 9) versus low (columns 8 and 10) state centralization LGAs for 6-9 and 34-39 cohorts while exclude state centralization and interaction variables from the regression model.

^a^ Significant at 1% level.

^b^ Significant at 1% level.

^c^ Significant at 1% level.

First, we want to ensure that the permanent historical and geographical differences between northern and southern Nigeria are not confounding the reported results. To facilitate comparison, we show the beta coefficients in brackets while showing the normal standard error in parentheses. Therefore, we introduced an indicator variable to the north. Columns 1 and 2 report our regression results. All three key explanatory variables are statistically and economically significant.

Second, we ran regressions for the northern sample to ensure that the vast differences in the northern and southern regions were not driving results. In this case, we compare areas in the north with differential exposure to both historical state complexity and Koranic education investment. To a large extent, this should mitigate the role that unobservables might play in influencing the results. Columns (3) and (4) show the regression estimates. Again, all the key variables remain significant. This shows that the difference between north and south cannot account for our reported results. Restricting regressions to a pair of LGAs within 200 km of each other yielded similar results to the previous regressions ^7^ (Columns 5 and 6). The empirical estimates reported in columns 7-10 showed where that Koranic education has the greatest effect on education outcomes. This is done by splitting LGAs into two groups, one group having to 3-4 layers of governmental authority beyond the local level (high state centralization LGAs) and the other having to 1-2 layers of authority (low-state centralization LGAs). For the two cohorts (6-9 and 34-39), estimates of the effects of Koranic education on school participation are negative and statistically significant. However, the effects in high-state centralization LGAs are nearly four times the effect on low-state centralization LGAs (Columns 7-10).

Finally, we implemented the Instrumental Variable (IV) method, which exploits the fact that Koranic education was ongoing in areas where Christian missionaries were restricted from reaching by the colonial government, and not necessarily because these areas were unreachable ( Okoye and Pongou (2024). Therefore, we used the Emirate dummy variable as an instrument for Koranic education, assigning 1 to LGAs considered restricted areas for missionaries ^8^. Table 5 presents the regression results. IV regressions are reported for the 6-9 and 34-39 cohorts for all samples, as well as high- and low-state centralization LGAs (Column 1-6). Koranic education had negative and significant effects on school enrolment for both cohorts and the whole sample, but significantly more so for high-state centralization areas. IV regressions for the northern sample implemented for high- and low-state centralization LGAs produce similar results. Koranic education tends to have greater negative effects on school enrolment in high-state centralization LGAs than in low-state centralization LGAs (Columns 7-10). These results should only be regarded as suggestive of causal effects because we do not have definitive evidence to make any serious claims about causal inference.

**Table 5.** Dependent Variable: School Enrolment (IV Regressions): More Robustness Checks.

Panel A 6-9 Cohorts	6-9 Cohorts	6-9 Cohorts	6-9 Cohorts	34-39 Cohorts	34-39 Cohorts	34-39 Cohorts	6-9 Cohorts	6-9 Cohorts	34-39 Cohorts	34-39 Cohorts
Sample	Whole Sample	High SC	Low SC	Whole Sample	High SC	Low SC	North + High SC	North + Low SC	North + High SC	North + Low SC
Second Stage										
Koranic Education	-45.356 ^a^ (5.6755)	-58.625 ^a^ (6.1134)	-18.046 ^a^ (6.3650)	-40.891 ^a^ (7.2208)	-52.266 ^a^ (10.3562)	-17.088 ^c^ (8.6464)	-46.021 ^a^ (8.5475)	-12.174 ^c^ (6.7017)	-52.332 ^a^ (16.3140)	-14.119 (10.8012)
F-Statistics	34.10	24.59	9.76	45.25	33.72	11.60	6.63	4.76	9.01	5.03
Observations	769	554	215	681	502	179	248	168	212	135
First Stage	*Koranic Education*									
Emirate	0.4833 ^a^ (0.0201)	0.4714 ^a^ (0.0247)	0.4749 ^a^ (0.0429)	0.4725 ^a^ (0.0209)	0.4402 ^a^ (0.0250)	0.4723 ^a^ (0.0487)	0.2940 ^a^ (0.0413)	0.4309 ^a^ (0.0511)	0.2409 ^a^ (0.0422)	0.4488 ^a^ (0.0595)

OLS method is used in the regressions reported here. Standard errors indicated in parenthesis are clustered at the state level. District (or LGA) is the unit of observation. CS is the enrolment rate for children who in 2006 were in the 6-9 age category. In columns 1-3, we ran for 6-9 cohorts and from 4-6 for 34-39 cohorts. We exclude state centralization and interaction variables from the regression model.

^a^ Significant at 1% level.

^b^ Significant at 1% level.

^c^ Significant at 1% level.

## 6. Conclusion

This study investigates the long-term influence of precolonial state centralization and Koranic education on school participation in contemporary Nigeria, with a focus on enrolment in government-led, tuition-free education programs such as the 1976 Universal Primary Education (UPE) and the 1999 Universal Basic Education (UBE). Our findings indicate that precolonial centralization is positively associated with school enrolment; however, this positive relationship is significantly attenuated—or even reversed—in districts where there was a high historical investment in Koranic education. These results are robust across multiple specifications and persist after controlling for a comprehensive set of historical and geographic variables. While our analysis includes an instrumental variable approach using Emirate region status as an instrument for Koranic education, I remain cautious in interpreting these estimates due to limitations in instrument strength and validity. Thus, I do not claim causal inference, and interpret our findings as robust correlations that reflect the enduring legacy of institutional and cultural history.

The study adds to the broader literature on institutional persistence, reversal of fortune, and the cultural determinants of human capital development. Specifically, it highlights how beneficial features of historical state capacity can be undermined by parallel cultural forces, such as deeply embedded preferences for Islamic education that may conflict with modern, secular schooling. The case of Northern Nigeria demonstrates how historical exposure to centralized authority does not uniformly translate into development gains, particularly where social norms discourage participation in formal education systems. These insights have important implications for education policy. Interventions aimed at increasing enrolment must account for cultural resistance and local institutional histories. Blending formal curricula with locally accepted religious frameworks, increasing community engagement, and aligning incentives with local values may be more effective than infrastructure expansion alone.

However, several limitations must be acknowledged. First, although the study explores historical determinants of educational outcomes, it does not make strong causal claims. The primary empirical strategy relies on OLS regressions, and while instrumental variable (IV) approaches were explored in a limited capacity, the results are interpreted with caution. The absence of natural experiments or clearly defined treatment and control groups also limits the applicability of quasi-experimental methods such as difference-in-differences (DiD) or propensity score matching (PSM).

Second, while the study controls for a wide range of historical and geographic factors, including 37 state fixed effects to account for time-invariant state-level heterogeneity, it does not directly test alternative mechanisms such as contemporary economic conditions, labor market incentives, or broader cultural norms beyond Koranic education. These are important areas for future research and should be considered when interpreting the findings.

In addition, the study suggests several directions for future research. Longitudinal data and quasi-experimental designs such as difference-in-differences (DiD) or regression discontinuity designs (RDD) could help establish causal mechanisms. Greater attention should also be given to how cultural traits such as religious norms, gender roles, and social sanctions influence education choices over time. As Nigeria and other countries in Sub-Saharan Africa continue to invest in human capital, understanding the historical and cultural context remains critical for designing inclusive and effective policies.

Finally, the policy implications of this study point to the need for more context-sensitive educational interventions. In regions with strong traditions of Koranic education, integrating formal schooling with culturally relevant curricula, improving community engagement, and addressing institutional trust deficits may be more effective than infrastructure expansion alone. Policymakers should consider these historical and cultural dynamics when designing inclusive education strategies.

## Ethics and consent

Ethics and consent are not required.

## Data Availability

The data was obtained from multiple sources and would be made available to other researchers after the study has been published. All data sources are cited in the study. Supplementary Material can be found at: https://www.doi.org/10.6084/m9.figshare.28130474

## Footnotes

^1^
Detailed discussion is contained in Appendix Section B.

^2^
The data from obtained from Nunn and Wantchekon (2011).

^3^
These distance variables are measured in metres. These are all from GeoAid.

^4^
Both are measures of determining the influence of outlying observations by measuring the difference in the estimated coefficients of key variables when the observations are added and when they are omitted from the regression sample.

^5^
RREG and QREG are robust and quantile regressions respectively, they produce regression estimates robust to the presence of influential observations in the regression sample.

^6^
Detailed discussion is contained in section C of the appendix.

^7^
In fact, running regressions with pair of LGAs within less than 150km, 100km or 50kmof each other produce qualitatively similar results. Results available upon request.

^8^
This includes all LGAs in states of the north-east and north-west except Adamawa and Taraba ( Okoye and Pongue, 2024).
